# Supplementary material for: Optimization and Application of Real-Time qPCR Assays in Detection and Identification of Chlamydiales in Products of Domestic Ruminant Abortion
Source: Pathogens. 2023 Feb 9;12(2):290. doi: 10.3390/pathogens12020290 (PMC9965055; doi:10.3390/pathogens12020290)
Supplement: Supplementary file 1 [file pathogens-12-00290-s001.zip › pathogens-2148273-supplementary/Supplementary files/Table S12_Results of analysis of samples without placentitis or pneumonia.pdf]

Table S12. Testing of foetal tissue/placenta of known uninfected animals to demonstrate specificity. (Samples from uninfected animals was defined as samples from abortion cases where no typical pathological lesions were noted).

No.	Primer/Probe					
	**Pan-Chlamydiales**	**Lab. number**	**Animal**	**Pathological lesion**	**Ct**	**Ct**
1	Placenta	B01488-18	Ovine	None	34.57	35.23
2	Placenta	B01800-18	Ovine	None	34.24	33.08
3	Placenta	B00920-19	Ovine	None	30.78	29.28
4	Placenta	B01010-19	Ovine	Generalised congestion	37.00	34.94
5	Placenta	B02033-19	Ovine	None	33.55	32.43
6	Stomach content	B01800-18	Ovine	None	37.12	ND
7	Stomach content	B02302-19	Ovine	Non-specific	ND	ND
8	Lung	B0215-18	Ovine	None	ND	ND
9	Lung	B01800-18	Ovine	None	ND	ND
10	Lung	B00902-19	Ovine	None	ND	ND
11	Lung	B01010-19	Ovine	Generalised congestion	12.40	ND
12	Lung	B02033-19	Ovine	None	ND	ND
13	Placenta	B01765-18	Bovine	None	8.35	ND
14	Placenta	B02311-18	Bovine	None	33.95	ND
15	Placenta	B01167-19	Bovine	None	34.51	32.62
16	Placenta	B01309-19	Bovine	None	35.56	36.71
17	Stomach content	B01259-18	Bovine	None	ND	ND
18	Stomach content	B01355-18	Bovine	None	36.26	ND
19	Stomach content	B01457-18	Bovine	None	ND	ND
20	Stomach content	B01498-18	Bovine	None	ND	ND
21	Stomach content	B01499-18	Bovine	None	21.92	ND
22	Stomach content	B01631-18	Bovine	Splenic necrosis	ND	ND
23	Stomach content	B01011-19	Bovine	None	32.93	33.75
24	Stomach content	B01431-19	Bovine	None	36.67	ND
25	Stomach content	B01482-19	Bovine	Epicardial ecchimoses, subcut haemorrhages, anasarca	ND	ND
26	Lung	B02311-18	Bovine	None	ND	21.12
27	Lung	B01457-18	Bovine	None	11.87	ND
28	Lung	B01167-19	Bovine	None	32.82	31.71
29	Lung	B01860-19	Bovine	None	3.54	35.49
30	Lung	B02063-19	Bovine	Nodular liver	18.18	12.44
31	Lung	B00490-18	Bovine	Pulmonary leukostasis	ND	ND
32	Lung	B00883-19	Bovine	Yellow liver	ND	ND
33	Placenta	B00771-18	Caprine	None	36.92	36.89

34	Placenta	B01567-18	Caprine	Lung haemorrhages, Hepatic necrosis	ND	ND
35	Placenta	B01582-18	Caprine	None	ND	ND
36	Placenta	B01756-19	Caprine	None	17.84	ND
37	Placenta	B01980-19	Caprine	Brain congestion, pericardial effusion	33.08	31.57
38	Stomach content	B01638-18	Caprine	Abomasitis, Vascular leucostasis	34.13	34.60
39	Stomach content	B0876-19	Caprine	None	ND	ND
40	Stomach content	B1456-19	Caprine	None	ND	12.82
41	Stomach content	B01756-19	Caprine	None	ND	ND
42	Stomach content	B02378-19	Caprine	Pleuritis, Hydropericardium	ND	ND
43	Stomach content	B01756019	Caprine	None	ND	ND
44	Lung	B01567-18	Caprine	Lung haemorrhages, Hepatic necrosis	ND	ND
45	Lung	B01582-18	Caprine	None	39.05	ND
46	Lung	B01638-18	Caprine	Abomasitis, Vascular leucostasis	ND	ND
47	Lung	B01456-19	Caprine	None	ND	ND
48	Lung	B02245-19	Caprine	Bacteraemia	36.63	5.87
49	Lung	B02378-19	Caprine	Pleuritis, Hydropericardium	ND	ND
	Chlamydia abortus	**Lab. number**	**Animal**	**Pathological lesion**	**Ct**	**Ct**
1	Placenta	B01488-18	Ovine	None	ND	ND
2	Placenta	B01800-18	Ovine	None	ND	32.23
3	Placenta	B00920-19	Ovine	None	ND	ND
4	Placenta	B01010-19	Ovine	Generalised congestion	ND	ND
5	Placenta	B02033-19	Ovine	None	9.26	7.17
6	Stomach content	B01800-18	Ovine	None	ND	ND
7	Stomach content	B02302-19	Ovine	Non-specific	ND	ND
8	Lung	B0215-18	Ovine	None	ND	ND
9	Lung	B01800-18	Ovine	None	ND	ND
10	Lung	B00902-19	Ovine	None	19.40	ND
11	Lung	B01010-19	Ovine	Generalised congestion	13.14	ND
12	Lung	B02033-19	Ovine	None	ND	ND
13	Placenta	B01765-18	Bovine	None	ND	ND
14	Placenta	B02311-18	Bovine	None	ND	ND
15	Placenta	B01167-19	Bovine	None	ND	ND
16	Placenta	B01309-19	Bovine	None	ND	14.07

17	Stomach content	B01259-18	Bovine	None	29.90	ND
18	Stomach content	B01355-18	Bovine	None	ND	ND
19	Stomach content	B01380-18	Bovine	None	33	ND
20	Stomach content	B01457-18	Bovine	None	ND	ND
21	Stomach content	B01498-18	Bovine	None	ND	ND
22	Stomach content	B01499-18	Bovine	None	ND	5.54
23	Stomach content	B01631-18	Bovine	Splenic necrosis	ND	ND
24	Stomach content	B01011-19	Bovine	None	ND	ND
25	Stomach content	B01431-19	Bovine	None	ND	4.49
26	Stomach content	B01482-19	Bovine	Epicardial ecchimoses, subcut haemorrhages, anasarca	ND	ND
27	Lung	B00470-18	Bovine	Pulmonary leukostasis	ND	ND
28	Lung	B02311-18	Bovine	None	10.21	ND
29	Lung	B01167-19	Bovine	None	3.68	ND
30	Lung	B01457-19	Bovine	None	ND	ND
31	Lung	B01498-19	Bovine	None	ND	14.52
32	Lung	B01860-19	Bovine	None	15	ND
33	Lung	B02063-19	Bovine	Nodular liver	35.79	ND
34	Placenta	B00771-18	Caprine	None	ND	ND
35	Placenta	B01567-18	Caprine	Lung haemorrhages, Hepatic necrosis	ND	ND
36	Placenta	B01582-18	Caprine	None	ND	5.13
37	Placenta	B01756-19	Caprine	None	4.91	8.72
38	Placenta	B02593-19	Caprine	Brain congestion, pericardial effusion	ND	ND
39	Stomach content	B01638-18	Caprine	Abomasitis, Vascular leucostasis	35.10	ND
40	Stomach content	B0876-19	Caprine	None	13.02	ND
41	Stomach content	B1456-19	Caprine	None	ND	ND
42	Stomach content	B02378-19	Caprine	Pleuritis, Hydropericardium	ND	5.64
43	Stomach content	B01756-19	Caprine	None	ND	ND
44	Lung	B01567-18	Caprine	Lung haemorrhages, Hepatic necrosis	ND	ND
45	Lung	B01582-18	Caprine	None	3.14	ND
46	Lung	B01638-18	Caprine	Abomasitis, Vascular leucostasis	16.72	ND
47	Lung	B01456-19	Caprine	None	ND	ND
48	Lung	B02245-19	Caprine	Bacteraemia	ND	ND
49	Lung	B02378-19	Caprine	Pleuritis, Hydropericardium	ND	ND

	Chlamydia pecorum	**Lab. number**	**Animal**	**Pathological lesion**	**Ct**	**Ct**
1	Placenta	B01488-18	Ovine	None	ND	ND
2	Placenta	B01800-18	Ovine	None	ND	ND
3	Placenta	B00920-19	Ovine	None	ND	ND
4	Placenta	B01010-19	Ovine	Generalised congestion	ND	ND
5	Placenta	B02033-19	Ovine	None	ND	ND
6	Stomach content	B01800-18	Ovine	None	ND	ND
7	Stomach content	B02302-19	Ovine		ND	ND
8	Lung	B0215-18	Ovine	None	ND	ND
9	Lung	B01800-18	Ovine	None	ND	ND
10	Lung	B00902-19	Ovine	None	ND	ND
11	Lung	B01010-19	Ovine	Generalised congestion	ND	ND
12	Lung	B02033-19	Ovine	None	ND	ND
13	Placenta	B01765-18	Bovine	None	ND	ND
14	Placenta	B02311-18	Bovine	None	ND	ND
15	Placenta	B01167-19	Bovine	None	ND	ND
16	Placenta	B01309-19	Bovine	None	ND	ND
17	Stomach content	B01259-18	Bovine	None	ND	ND
18	Stomach content	B01355-18	Bovine	None	ND	ND
19	Stomach content	B01380-18	Bovine	None	ND	ND
20	Stomach content	B01457-18	Bovine	None	ND	ND
21	Stomach content	B01498-18	Bovine	None	ND	ND
22	Stomach content	B01499-18	Bovine	None	ND	ND
23	Stomach content	B01631-18	Bovine	Splenic necrosis	ND	ND
24	Stomach content	B1011-19	Bovine	None	ND	ND
25	Stomach content	B01431-19	Bovine	None	ND	ND
26	Stomach content	B01482-19	Bovine	Epicardial ecchimoses, subcut haemorrhages, anasarca	ND	ND
27	Lung	B0490-18	Bovine	Pulmonary leukostasis	ND	ND
28	Lung	B00883-19	Bovine	Yellow lung	ND	ND
29	Lung	B01167-19	Bovine	None	ND	ND
30	Lung	B01457-18	Bovine	None	ND	ND
31	Lung	B01860-19	Bovine	None	ND	ND
32	Lung	B02063-19	Bovine	Nodular liver	ND	ND
33	Lung	B02311-18	Bovine	None	ND	ND
34	Placenta	B00771-18	Caprine	None	ND	ND

35	Placenta	B01567-18	Caprine	Lung haemorrhages, Hepatic necrosis	ND	ND
36	Placenta	B01582-18	Caprine	None	ND	ND
37	Placenta	B01756-19	Caprine	None	ND	ND
38	Placenta	B01980-19	Caprine	Brain congestion, pericardial effusion	ND	ND
39	Stomach content	B01638-18	Caprine	Abomasitis, Vascular leucostasis	ND	ND
40	Stomach content	B0876-19	Caprine	None	ND	ND
41	Stomach content	B1456-19	Caprine	None	ND	ND
42	Stomach content	B02378-19	Caprine	Pleuritis, Hydropericardium		
43	Stomach content	B01756-19	Caprine	None	ND	ND
44	Lung	B01567-18	Caprine	Lung haemorrhages, Hepatic necrosis	ND	ND
45	Lung	B01582-18	Caprine	None	ND	ND
46	Lung	B01638-18	Caprine	Abomasitis, Vascular leucostasis	ND	ND
47	Lung	B01456-19	Caprine	None	ND	ND
48	Lung	B02245-19	Caprine	Bacteraemia	ND	ND
49	Lung	B02378-19	Caprine	Pleuritis, Hydropericardium	ND	ND
	Parachlamydia acanthamoeba	**Lab. number**	**Animal**	**Pathological lesion**	**Ct**	**Ct**
1	Placenta	B01488-18	Ovine	None	36.49	36.67
2	Placenta	B01800-18	Ovine	None	36.60	35.44
3	Placenta	B00920-19	Ovine	None	35.53	ND
4	Placenta	B01010-19	Ovine	Generalised congestion	35.65	37.02
5	Placenta	B02033-19	Ovine	None	36.89	34.98
6	Stomach content	B01800-18	Ovine	None	35.75	36.95
7	Stomach content	B02302-19	Ovine	Non-specific	ND	ND
8	Lung	B0215-18	Ovine	None	36.78	36.07
9	Lung	B01800-18	Ovine	None	35.50	35.88
10	Lung	B00902-19	Ovine	None	34.93	34.10
11	Lung	B01010-19	Ovine	Generalised congestion	36.69	37.02
12	Lung	B02033-19	Ovine	None	35.92	35.61
13	Placenta	B01765-18	Bovine	None	34.36	35.84
14	Placenta	B02311-18	Bovine	None	34.54	35.22
15	Placenta	B01167-19	Bovine	None	35.79	ND
16	Placenta	B01309-19	Bovine	None	35.99	37.05

17	Stomach content	B01259-18	Bovine	None	33.78	30.99
18	Stomach content	B01355-18	Bovine	None	35.49	33.56
19	Stomach content	B01457-18	Bovine	None	35.32	35.85
20	Stomach content	B01498-18	Bovine	None	35.04	33.07
21	Stomach content	B01499-18	Bovine	None	36.33	36.94
22	Stomach content	B01631-18	Bovine	Splenic necrosis	ND	35.51
23	Stomach content	B1011-19	Bovine	None	35.86	36.12
24	Stomach content	B01431-19	Bovine	None	36.39	ND
25	Stomach content	B01482-19	Bovine	Epicardial ecchimoses, subcut haemorrhages, anasarca	36.53	35.92
26	Lung	B00490-18	Bovine	Pulmonary leukostasis	36.78	36.07
27	Lung	B00883018	Bovine	Yellow liver	34.93	34.10
28	Lung	B02311-18	Bovine	None	35.88	35.33
29	Lung	B01167-19	Bovine	None	36.83	36.11
30	Lung	B01457-18	Bovine	None	35.32	35.85
31	Lung	B01496-19	Bovine	Viral placentitis	34.94	36.05
32	Lung	B01860-19	Bovine	None	35.42	36.45
33	Lung	B02063-19	Bovine	Nodular liver	34.98	34.88
34	Placenta	B00771-18	Caprine	None	35.82	36.07
35	Placenta	B01567-18	Caprine	Lung haemorrhages, Hepatic necrosis	ND	35.57
36	Placenta	B01582-18	Caprine	None	ND	ND
37	Placenta	B01756-19	Caprine	None	33.27	36.05
38	Placenta	B02593-19	Caprine	Brain congestion, pericardial effusion	35.98	36.13
39	Stomach content	B01638-18	Caprine	Abomasitis, Vascular leucostasis	36.43	36.31
40	Stomach content	B0876-19	Caprine	None	36.79	36.12
41	Stomach content	B1456-19	Caprine	None	36.07	35.35
42	Stomach content	B02378-19	Caprine	Pleuritis, Hydropericardium	35.89	35.97
43	Stomach content	B01756-19	Caprine	None	32.75	35.57
44	Lung	B01567-18	Caprine	Lung haemorrhages, Hepatic necrosis	35.92	ND
45	Lung	B01582-18	Caprine	None	ND	35.58
46	Lung	B01638-18	Caprine	Abomasitis, Vascular leucostasis	35.36	35.46
47	Lung	B01456-19	Caprine	None	36.10	35.96
48	Lung	B02245-19	Caprine	Bacteraemia	35.83	36.03
49	Lung	B02378-19	Caprine	Pleuritis, Hydropericardium	34.78	36.92

	Waddlia chondrophila	Lab. number	Animal	Pathological lesion	Ct	Ct
1	Placenta	B01488-18	Ovine	None	ND	ND
2	Placenta	B01800-18	Ovine	None	ND	ND
3	Placenta	B00920-19	Ovine	None	ND	ND
4	Placenta	B01010-19	Ovine	Generalised congestion	ND	ND
5	Placenta	B02033-19	Ovine	None	ND	ND
6	Stomach content	B01800-18	Ovine	None	ND	ND
7	Stomach content	B02302-19	Ovine	Non-specific	ND	ND
8	Lung	B0215-18	Ovine	None	ND	ND
9	Lung	B01800-18	Ovine	None	ND	ND
10	Lung	B00902-19	Ovine	None	ND	ND
11	Lung	B01010-19	Ovine	Generalised congestion	ND	ND
12	Lung	B02033-19	Ovine	None	ND	ND
13	Placenta	B01765-18	Bovine	None	ND	ND
14	Placenta	B02311-18	Bovine	None	ND	ND
15	Placenta	B01167-19	Bovine	None	ND	ND
16	Placenta	B01309-19	Bovine	None	ND	ND
17	Stomach content	B01259-18	Bovine	None	ND	ND
18	Stomach content	B01355-18	Bovine	None	ND	ND
19	Stomach content	B01457-18	Bovine	None	34.29	ND
20	Stomach content	B01498-18	Bovine	None	ND	ND
21	Stomach content	B01499-18	Bovine	None	ND	ND
22	Stomach content	B01631-18	Bovine	Splenic necrosis	ND	ND
23	Stomach content	B01011-19	Bovine	None	ND	ND
24	Stomach content	B01431-19	Bovine	None	ND	ND
25	Stomach content	B01482-19	Bovine	Epicardial ecchimoses, subcut haemorrhages, anasarca	ND	ND
26	Lung	B00490-18	Bovine	Pulmonary leukostasis	ND	ND
27						
28	Lung	B02311-18	Bovine	None	ND	ND
29	Lung	B01167-19	Bovine	None	ND	ND
30	Lung	B01457-18	Bovine	None	ND	ND
31	Lung	B01498-19	Bovine	None	ND	ND
32	Lung	B01860-19	Bovine	None	ND	ND
33	Lung	B02063-19	Bovine	Nodular liver	ND	ND
34	Placenta	B00771-18	Caprine	None	ND	ND
35	Placenta	B01567-18	Caprine	Lung haemorrhages, Hepatic necrosis	ND	ND

36	Placenta	B01582-18	Caprine	None	ND	ND
37	Placenta	B01756-19	Caprine	None	ND	ND
38	Placenta	B01980-19	Caprine	Brain congestion, pericardial effusion	ND	ND
39	Stomach content	B01638-18	Caprine	Abomasitis, Vascular leucostasis	ND	ND
40	Stomach content	B0876-19	Caprine	None	ND	ND
41	Stomach content	B1456-19	Caprine	None	ND	ND
42	Stomach content	B02378-19	Caprine	Pleuritis, Hydropericardium	ND	ND
43	Stomach content	B01756-19	Caprine	None	ND	ND
44	Lung	B01567-18	Caprine	Lung haemorrhages, Hepatic necrosis	ND	ND
45	Lung	B01582-18	Caprine	None	ND	ND
46	Lung	B01638-18	Caprine	Abomasitis, Vascular leucostasis	ND	ND
47	Lung	B01456-19	Caprine	None	ND	ND
48	Lung	B02245-19	Caprine	Bacteraemia	ND	ND
49	Lung	B02378-19	Caprine	Pleuritis, Hydropericardium	ND	ND
