# Supplementary material for: Optimization and Application of Real-Time qPCR Assays in Detection and Identification of Chlamydiales in Products of Domestic Ruminant Abortion
Source: Pathogens. 2023 Feb 9;12(2):290. doi: 10.3390/pathogens12020290 (PMC9965055; doi:10.3390/pathogens12020290)
Supplement: Supplementary file 1 [file pathogens-12-00290-s001.zip › pathogens-2148273-supplementary/Supplementary files/Table S1_Sequences of synthetic controls.pdf]

Table S1. Sequences (5′ to 3′) of synthetic controls used for the optimisation of the Pan-Chlamydiales assay and the *C. abortus*/*C. pecorum* and *P. acanthamoeba*/*W. chondrophila* assays.

**Synthetic controls**	**Sequence (5′ to 3′)**
Pan-Chlamydiales assay	CCG CCA ACA CTG GGA CTG AGA CAC TGC CCA GAC TTC TAC GGA AGG CTG CAG TCG AGA ATC TTT CGC AAT GGA CGA AAG TCT GAC GAA GCG ACG CCG CGT GTG TGA TGA AGG CTC TAG GGT TGT AAA GCA CTT TCG CTT GGG AAT AAG AGA GAT TGG CTA ATA TCC AAT CGA TTT GAG CGT ACC AGG TAA AGA AGC ACC GGC TAA CTC C
C. abortus/*C. pecorum* assay<br>*P. acanthamoeba*/*W. chondrophila* assay	CTC AAC TCC AGA ACA GCA TTT GAA ACT ATA TTT CTT GAG GGT AGG CGG AGA AAA CGG AAT TCC ACA TGT AGC GGT GAA ATG CGT AGA TAT GTG GAA GAA CAT CGG TGG CGA AGG CGG TTT TCT AGC TTA TTC CTG ACG CTG AGA CTA GGC CCT TGG GTC GTA AAG TTC TTT CGC ATG GGA ACA AGA GAA GGA TGC TAA TAT CAT CTG GAT TTG AGC GTA CCT TGT AAA GAA GCA CCG GCT AAC TCC GAC TAG CAA CTG ACA CTA AGT CGG CTA CAA TTA AAT ACC ACG AAT GGC AAG TTG GTT TAG CGC TCT CTT ATC GAT TGA ACA TGC TTG TAC TAC CAT GTG ATC CTT GCG CTA CTT GGT GCG ACG CGA TTA GCT TAC GCG TAG GAT TTT ACG GAG ATT ATG TTT TCG ACA
