# Supplementary material for: Optimization and Application of Real-Time qPCR Assays in Detection and Identification of Chlamydiales in Products of Domestic Ruminant Abortion
Source: Pathogens. 2023 Feb 9;12(2):290. doi: 10.3390/pathogens12020290 (PMC9965055; doi:10.3390/pathogens12020290)
Supplement: Supplementary file 1 [file pathogens-12-00290-s001.zip › pathogens-2148273-supplementary/Supplementary files/Tables S2-S11_Limit of detection data.pdf]

Supplementary tables – Limit of detection

Sensitivity: Synthetic controls: Limit of detection in placenta, abomasal fluid and lung. Testing foetal tissues and placenta spiked with the synthetic DNA fragment of interest.

Pan-Chlamydiales qPCR

Table S2. Results of analysis of spiked placenta DNA.

Sample: Placenta	
**DNA copies**	**C_T (Target: Pan-Chlamydiales)**
500	34,74
500	34,79
500	34,52
500	34,32
500	34,79
500	36,89
250	31,08
250	30,86
250	30,72
250	30,81
250	30,83
250	30,90
125	30,46
125	30,67
125	30,21
125	30,60
125	30,72
125	32,04
62,5	30,27
62,5	30,13
62,5	29,99
62,5	30,27
62,5	30,51
62,5	30,52

Table S3. Results of analysis of spiked stomach content DNA.

Sample: Stomach content	
**DNA copies**	**C_T (Target: Pan-Chlamydiales)**
500	35,15
500	35,96
500	34,35
500	35,26
500	35,27

500	Undetermined
250	36,47
250	36,06
250	35,38
250	Undetermined
250	35,59
250	35,44
125	36,08
125	35,39
125	35,23
125	35,94
125	35,15
125	35,25
62,5	35,51
62,5	35,91
62,5	35,58
62,5	35,60
62,5	33,99
62,5	36,47

Table S4. Results of analysis of spiked lung DNA.

**Limit of detection: Lung**	
**DNA copies**	**C_T (Target: Pan-Chlamydiales)**
1000	32,12
1000	32,38
1000	32,17
1000	32,61
1000	32,36
1000	32,47
500	32,49
500	32,61
500	33,17
500	33,58
500	33,81
500	33,64
250	34,14
250	35,43
250	35,19
250	33,94
250	34,99
250	34,98
125	36,00
125	35,96
125	35,27

125	36,01
125	36,02
125	35,92

Limit of detection*: C abortus*/*C pecorum* assay

Table S5. Results of analysis of spiked placenta DNA.

Sample: Placenta		
**DNA copies**	**CT (Target: *C. abortus*)**	**CT (Target: *C pecorum*)**
10	33,31	32,94
10	33,73	33,10
10	32,70	32,28
10	32,79	32,34
10	33,52	32,87
10	33,76	33,18
5	34,22	33,51
5	35,28	34,53
5	33,62	32,74
5	34,43	33,38
5	37,05	35,08
5	Undetermined	Undetermined
2	34,51	34,04
2	35,28	34,42
2	33,84	33,39
2	35,69	34,98
2	Undetermined	Undetermined
2	Undetermined	Undetermined
1	37,51	36,94
1	35,18	34,69
1	36,79	34,79
1	35,95	33,95
1	37,27	36,56
1	35,82	33,90

Table S6. Results of analysis of spiked stomach content DNA.

**Sample: Stomach content**		
**DNA copies**	**CT (Target: *C. abortus*)**	**CT (Target: *C. pecorum*)**
10	36,49	35,58
10	33,57	32,99
10	34,39	34,55

10	35,60	36,60
10	34,75	34,63
10	35,49	34,96
5	34,04	34,07
5	34,07	33,21
5	34,72	33,68
5	37,10	36,25
5	Undetermined	37,14
5	34,97	33,93
2	34,74	34,75
2	Undetermined	Undetermined
2	Undetermined	Undetermined
2	35,87	35,23
2	35,63	34,33
2	34,85	33,80
1	35,92	35,53
1	34,67	34,02
1	36,39	35,94
1	36,99	36,29
1	35,63	36,64
1	36,30	35,17

Table S7. Results of analysis of spiked lung DNA.

**Tissue DNA: Lung**		
**DNA copies**	**Cт (Target: *C abortus*)**	**Cт (Target: *C. pecorum*)**
10	32,79	32,53
10	32,28	31,86
10	32,96	32,28
10	34,91	34,82
10	34,36	34,09
10	33,36	33,73
5	34,61	33,89
5	34,27	34,41
5	34,58	34,73
5	33,99	34,10
5	34,68	34,43
5	34,51	33,68
2	34,65	34,35
2	35,61	34,54
2	17,11	32,34
2	35,21	35,68
2	34,65	34,08

2	37,74	37,16
1	Undetermined	35,56
1	35,85	34,60
1	Undetermined	Undetermined
1	34,56	35,63
1	Undetermined	35,82
1	35,17	37,492

***Parachlamydia/Waddlia* qPCR assay**

Table S8. Results of analysis of spiked placenta DNA.

DNA copies (log)	C_T (Target: Parachlamydia)	C_T (Target Waddlia)
10	35,18	33,01
10	33,96	35,15
10	Undetermined	34,00
10	34,30	33,72
10	36,23	33,67
10	36,74	33,72
5	33,96	34,50
5	34,58	34,81
5	34,83	34,04
5	34,66	34,84
5	33,78	34,31
5	33,72	34,41
2	34,87	36,34
2	35,80	35,61
2	35,34	36,31
2	34,37	35,64
2	34,91	34,33
2	34,72	34,67
1	34,75	36,26
1	34,44	35,41
1	35,44	35,65
1	36,41	36,17
1	34,35	36,87
1	35,66	Undetermined

Table S9. Results of analysis of spiked stomach content DNA.

DNA copies (log)	Ct (Target *Parachlamydia*)	Ct (Target: *Waddlia*)
10	30,75	31,76
10	30,75	31,85
10	30,83	31,72
10	30,76	32,26
10	30,27	31,79
10	30,34	31,58
5	34,96	35,52
5	31,65	32,77
5	31,62	32,76
5	30,78	32,22
5	31,43	32,89
5	31,55	32,61
2	33,23	34,52
2	32,64	34,29
2	32,77	34,09
2	34,91	36,99
2	32,38	33,51
2	32,98	34,33
1	34,58	34,59
1	33,48	35,18
1	32,88	34,35
1	33,26	34,80
1	33,32	35,20
1	33,61	35,34

Table S10. Results of analysis of spiked lung DNA.

DNA copies (log)	Cт (Target: *Parachlamydia*)	Ct (Target: *Waddlia*)
10	27,90	28,75
10	27,87	28,76
10	27,59	28,60
10	27,67	28,75
10	27,79	28,59
10	27,90	28,49
5	30,85	29,62
5	31,17	29,16
5	30,57	29,16
5	30,04	28,69
5	30,73	29,48
5	30,17	29,83
2	31,51	30,02
2	30,96	30,78

2	31,19	31,11
2	30,28	30,73
2	30,33	30,68
2	32,14	30,85
1	33,32	31,78
1	31,68	31,44
1	31,62	30,52
1	38.00	31,70
1	32,18	32,24
1	32,74	32,40

Table S11. Summary of results of limit of detection analyses for the Pan-Chlamydiales, *C. abortus*/ *C. pecorum* and *P. acanthamoeba*/ *W. chondrophila* qPCR assays using extracted DNA of placenta, stomach content and lung spiked with decreasing concentrations of synthetic controls. Ca=*Chlamydia abortus*, Cp=*Chlamydia pecorum*, Pa=*Parachlamydia acanthamoeba*, Wc=*Waddlia chondrophila.*

**qPCR assay**	**Spiked DNA sample**		
	**Placenta**	**Stomach content**	**Lung**
	**DNA copies**	**DNA copies**	**DNA copies**
**Pan-Chlamydiales**	<62.5	<62.5	<125
C. abortus*/ *C. pecorum	5	5	2
P. acanthamoeba*/ *W. chondrophila	Pa: <1<br>Wc: 1	<1	<1
